# Forecasting of Airborne Conidia Quantities and Potential Insect Associations of *Cryphonectria parasitica*, the Causal Agent of Chestnut Blight, in England

**DOI:** 10.3390/jof10030181

**Published:** 2024-02-28

**Authors:** Pedro Romon-Ochoa, Pankajini Samal, Tom Pace, Tim Newman, Mark Oram, Nicholas Baxter, John A. S. Manning, Mick Biddle, Kerry Barnard, Daegan Inward, Paul Taylor, Steven Hendry, Ana Pérez-Sierra, Lisa Ward

**Affiliations:** 1 Forest Research, Plant Pathology Department, Alice Holt Research Station, Farnham GU10 4LH, Surrey, UK; pankajini.samal@forestresearch.gov.uk (P.S.); mick.biddle@forestresearch.gov.uk (M.B.); lisa.ward@forestresearch.gov.uk (L.W.); 2 Forest Research, TSU Exeter Field Station, Haldon Forest, Exeter EX6 7XR, UK; tom.pace@forestresearch.gov.uk (T.P.); tim.newman@forestresearch.gov.uk (T.N.); mark.oram@forestresearch.gov.uk (M.O.); 3 Forestry Commission, Plant Health Forestry Team, 620 Bristol Business Park, Bristol BS16 1EJ, UK; nicholas.baxter@forestrycommission.gov.uk (N.B.); john.manning@forestrycommission.gov.uk (J.A.S.M.); 4 Forest Research, Entomology Department, Alice Holt Research Station, Farnham GU10 4LH, Surrey, UK; kerry.barnard@forestresearch.gov.uk (K.B.); daegan.inward@forestresearch.gov.uk (D.I.); 5 Forest Research, Data and Statistics Department, Alice Holt Research Station, Farnham GU10 4LH, Surrey, UK; paul.taylor@forestresearch.gov.uk; 6 Forest Research, Tree Health Diagnostics and Advisory Service, Alice Holt, Farnham GU10 4LH, Surrey, UK; steven.hendry@forestresearch.gov.uk (S.H.); ana.perez-sierra@forestresearch.gov.uk (A.P.-S.)

**Keywords:** chestnut blight, conidia, detection, forecasting, insects, quantification

## Abstract

Sweet chestnut, an Asiatic tree introduced in many parts of Europe including the United Kingdom, is planted for nut production, timber, and amenity. Its major threat is the disease called blight, caused by the fungus *Cryphonectria parasitica*, which infects through wounds by airborne spores. Field trapping using sticky rods rotating traps was performed in an infected area in Devon (between May 2021 and April 2023). An improved dual hydrolysis Taqman probes real-time PCR was used. The number of spores was calculated by comparing the cycle threshold to the Ct of standards with known amounts of conidia or known target fragment copies cloned into a plasmid. Weekly spore counts were in the range of around 60 to approximately 8.5 × 10^3^, with fluctuations of peaks (mainly in late summer–autumn 2021) and troughs. The effects of weather parameters were modelled, finding correlations between spore numbers and temperature, humidity, dewpoint, rainfall, wind speed, and wind duration. Additionally, an insect trapping was performed to confirm the presence/absence and quantity of *C. parasitica* conidia potentially phoretic on some insects by using the same molecular approach. None of the ten collected insect species harboured spores of this fungus.

## 1. Introduction

Chestnut blight is a disease of *Castanea* species caused by the ascomycete *Cryphonectria parasitica* (Murrill) M. E. Barr., which can be considered as one of the major, if not the major, threat to sweet chestnut trees. The fungus originates from Eastern Asia [1], where it has little effect on the native host trees (Japanese chestnut *Castanea crenata* Sieb. and Zucc. and Chinese chestnut *Castanea mollisima* Meiling) [2].

It has caused, however, severe epidemics resulting in massive death and dieback of both *Castanea dentata* (Marshall) Borkh. in North America, where it was introduced in the late nineteenth century [3], and sweet chestnut (*Castanea sativa* Mill.) in most of continental Europe, where it was firstly introduced in Italy in 1938 [4]. Diseased trees exhibit crown dieback above girdling cankers on the trunk and/or branches with profuse epicormic growth below the cankers. Other symptoms are orange fruiting bodies that erupt through swollen lenticels and mycelial fans beneath the bark that spread into the cambium. Planting stock, timber, and bark are pathways of long-distance dispersal [5]. This pathogen is considered for its detrimental impact on forestry and agricultural orchards for nut production [6] and is a protected-zone quarantine pest in the EU according to the Commission Implementing Regulation 2019/2072.

The United Kingdom was considered free from chestnut blight until 2011, when infections were discovered on young saplings of sweet chestnut planted on a farm in Warwickshire, England [7]. All affected trees were destroyed. In 2013, the UK introduced controls on the import, movement, and export of sweet chestnut [8]. Until 2016, all findings of the disease (several nurseries in Devon, Herefordshire, Kent, Norfolk, Somerset, and East Sussex) were exclusively in orchards or recently planted young trees, and therefore their eradication, and subsequent monitoring around each site, was relatively easy. However, in December 2016, *C. parasitica* was detected on four mature trees in Devon, for the first time in the wider environment. Additional surveys revealed multiple introductions in south and mid England and in the Channel Islands of Jersey and Guernsey [9,10,11].

Research is now focused on possible biocontrol using Cryphonectria hypovirus 1 (CHV1) on those sites where one dominant Vegetative Compatibility Group (VCG) [12] is present, and CHV1 has been detected. CHV1 from highly infected fungal strains from Europe has been successfully transmitted to uninfected English *C. parasitica* isolates of the same VCGs (mainly EU10 and EU9) and further experiments about the *in planta* and thermal behaviour, under controlled conditions, of those virus-infected isolates have been completed successfully [13,14].

Regarding the reproduction of *C. parasitica*, two types of spores can be produced, asexual conidia and sexual ascospores, the latter depending on the simultaneous presence of both mating type alleles. Conidia are spread short distances (a few metres) by rain-splash and can be produced all year round under suitable conditions [15], while ascospores can be wind-dispersed longer distances, up to a few hundred metres [5]. No existence of sexual reproduction has been detected so far in Britain [9,10,16], except in the first detected nursery outbreak in 2011 [7].

Aerial spore concentrations are of importance in developing epidemiological models for most airborne diseases, for example, to discern the less appropriate seasons for doing sylvicultural works opening wounds (pruning, felling). Potential vector analyses are also important for indirect control management strategies. In this regard, the insect trapping in the current study was focused on *Xylosandrus germanus* (Blandford) (Coleoptera: Curculionidae, Scolytinae) since this ambrosia beetle has recently been loosely associated with the pathogen by Next Generation Sequencing (NGS), together with other 43 fungal taxa [17]. This beetle is highly polyphagous, has been introduced in Europe for several decades now, and it is currently considered an invasive species in forest ecosystems and plantations throughout Europe. Oriental Chestnut Gall Wasp (OCGW) *Dryocosmus kuriphilus* gall exit holes have been also documented as entry points for the chestnut blight fungus [18].

The objectives of this study were thus: (i) to develop a spore-trapping assay that can be used for extended periods, (ii) to determine the weekly quantity of *C. parasitica* airborne conidia in an affected plantation in Devon, (iii) to correlate the quantity of conidia with meteorological data including temperature, relative humidity, dew point, rain, wind speed, wind direction and duration, (iv) to develop an insect trapping assay especially targeting bark and ambrosia beetles, but also OCGW specimens (v) to confirm or rule out *C. parasitica* conidia potentially phoretic on those insects.

## 2. Materials and Methods

### 2.1. Spore and Insect Traps

Two rotorod spore traps (in-house-built according to SOP0186) were placed in an affected plantation (Devon) (50.629299, −3.46673) in north-south direction and 3 m apart from sporulating cankers. Traps were connected to a motorcycle 7 V battery that permitted continuous rotating for 48 h, and harboured two sticky rods that were collected weekly into the same sterile tube. An Easy Log USB-2 meteorological datalogger (Lascar Electronics, Salisbury, UK) was attached to each trap, to measure temperature (°C), relative humidity (%), and dew point (°C) every 20 min. Wind speed, direction and duration, and rain data were collected from the closest meteorological station (50.85969, −3.2389). Mean values were calculated for the week preceding each spore trap collection date. Correlation analyses were performed (Table 1).

To trap bark and ambrosia beetles, a total of 10 Lindgren seven-funnel traps with propylene glycol, the same number and type of traps, and 10 cross-vane mini traps were placed, respectively, at the same plantation in Devon, a park estate in West Sussex, and an urban park in East London, all sites with the presence of sporulating cankers of *C. parasitica*. Traps were suspended between trees with ropes (Lindgren in Devon and West Sussex), or in the lower canopy (cross-vane in London) to prevent public interference. The traps were baited to attract Scolytinae with 100% ethanol released from screw-top containers with holes drilled in the lid, to give a measured release rate of approximately 200 mg/day. Sometimes the traps were also operated dry (with tissue paper in the collecting jars instead of propylene glycol) for three days periods to capture a subsample of alive beetles. OCGW infested twigs harbouring fresh galls were also collected and included in emergence traps, rendering *Dryocosmus kuriphilus* (OCGW) and one unidentified species of the hyperparasitoid genus *Torymus*.

### 2.2. DNA Extraction from Sticky Rods

DNA was extracted following a semi-automated method. Spores were disrupted from the sticky rods by shaking with glass beads: 0.3 ± 0.02 g of 150–212 µm plus 0.1 ± 0.01 g of 425–600 µm glass beads (Merck, Feltham, UK), added to each sample tube using a scoop made with a 0.2 mL tube attached to a 1 mL Pasteur pipette (modified from Dvorak et al. [19]) and shaken for 40 s at 2700 rpm in a PowerLyzer 24 homogenizer (Qiagen, Manchester, UK). The tubes were kept on ice for 5 min, and then 220 µL of 0.1% Nonidet solution was added and another cycle was run under the same conditions. After keeping the tubes on ice for an additional 5 min, a third shaking cycle was performed, after which 350 µL of Lysis Buffer A (MAgMAX isolation kit, Applied Biosystems, Fisher Scientific, Horsham, UK) was added, and the tubes vortexed for 20 s. Then 50 µL of Lysis Buffer B and 20 µL RNase A were added to each tube and vortexed for 20 s. While incubating the tubes in a hot block at 65 °C for 10 min with occasional vortexing, five different plate types for a Kingfisher DNA extraction machine (Fisher Scientific, Horsham, UK) were prepared (Wash Buffer 1 deep well plate, Wash Buffer 2.1 deep well plate, Wash Buffer 2.2 deep well plate, Elution Buffer standard well plate, Tip Comb for DW Magnets in a standard well plate) following MAgMAX isolation Kit instructions.

Afterwards, 130 µL Precipitation Buffer was added and mixed by inverting each tube three times. After incubating on ice for 5 min, the lysate was cleared by centrifuging for 5 min at 20,000× *g*. One additional Kingfisher plate type was then prepared by adding to a deep well plate, 25 µL of magnetic beads, well-resuspended before use, and 400 µL of 96–100% ethanol, mixing by pipetting, before adding 400 µL of each previous sample supernatant. After turning on the Kingfisher machine, prompts were followed on screen to load the respective plates and, after approximately 36 min, the extracted DNA in the elution plate was covered with a standard plate adhesive seal and stored at −20 °C until used. This semi-automated method allowed processing up to 96 samples simultaneously.

### 2.3. Insects Processing

Insects were identified to species or genus level using appropiate taxonomical keys, with members of each species pooled together in tubes.

After counting the specimens in each tube (Supplementary Table S1), one millilitre of sterile 0.02% solution of Tween 80 was added to each tube and vortexed for 30 s to obtain washed insects sample. Two hundred microlitres of this washed insects sample were plated with a spreader onto two replicate PDA+S+A (39 g Potato Dextrose Agar with 0.05 g L^−1^ streptomycin and 0.25 g L^−1^ ampicillin) plates. The remaining 100 microlitres were used to prepare 1:10 and 1:100 dilution tubes with 900 µL Phosphate Buffer Saline (PBS (per litre), 8 g NaCl, 0.2 g KCl, 1.44 g Na_2_HPO_4_ and 0.24 g KH_2_PO_4_), plating 200 µL of each sample onto two PDA+S+A plates. After 5 days at 20 °C, the different fungal colonies were sub-cultured onto fresh PDA. Another 500 µL of this washing suspension was added to a tube containing a methallic bead for real-time PCR analysis.

The remaining insect specimens were cleaned by adding 1 mL PBS, vortexing for 15 s, and drying on a tissue paper. They were transferred to a new 1.5 mL tube, crushed with 200 µL PBS with the help of a sterile pestle and vortexed together with other 800 µL PBS, before preparing a dilution series for plating onto PDA+S+A agar as before. A separate metallic-bead-in tube containing 500 µL PBS of the homogenate was prepared.

Thus, two sets of tubes (external washing and washed-crushed insects) were made and processed by the real-time PCR explained below (Table 2), but using the DNeasy Plant Pro kit (Qiagen, Manchester, UK), following the manufacturer’s protocol, for DNA extractions from the metallic-bead-in tubes.

### 2.4. Real-Time PCR

A species-specific real-time PCR was performed using a modified and improved version of the TaqMan dual hydrolysis probe assay designed by Chandelier et al. [20]. PCRs were carried out on a LightCycler 480 (Roche). For the assay, 5 µL Takyon Blue (Eurogentec, Seraing, Belgium), 0.5 µL (final concentration 500 nM) of the primers Cp-F4 (5′-GATACCCTTTGTGAACTTATAA-3′), Cp-R3 (5′-GGGGAGAAGGAAGAAAATC-3′), 18SF (5′-GCAAGGCTGAAACTTAAAGGAA-3′), and 18SR (5′-CCACCACCCATAGAATCAAGA-3′), and 0.25 µL (final concentration 250 nM) of the probes Cp-S3 (5′-/6-FAM/TTTATCGTTGCCTCGGCGCTGA/BHQ1/-3′) and 18S probe (5′-/56-JOEN/ACGGAAGGGCACCACCAGGAGT/BHQ1/-3′), plus 2 µL molecular grade water and 0.5 µL DNA extract were used in a 10 µL reaction volume.

Conditions were 95 °C for 10 min followed by 40 cycles of 95 °C for 15 s and 60 °C for 1 min; all samples were tested in triplicate. Fluorescent detection occurred at the end of each 60 °C step. The cycle threshold (Ct) value was calculated automatically using the LightCycler software 4.1 with absolute quantification using second derivative maximum setting with 465–510 nm channel filter for analysing the specific amplicons and with 533–580 nm channel filter for analysing the 18S DNA quality control amplicons. Diluted 1:10, 1:100, 1:1000, and 1:10,000 positive controls were used. As positive control, a PlantPro kit DNA extract using four *C. parasitica* mycelial plugs was used.

**Figure 1:**
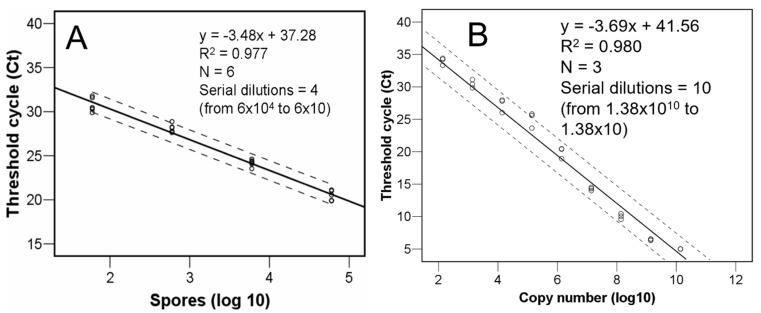
Equations relating *Cryphonectria parasitica* conidia (**A**) and ITS-5.8S-ITS2 target fragment copy numbers (**B**) with threshold cycle values. Dotted lines represent 95% confidence intervals.

The method was improved by using three replicates of ten 1:10 points serial dilution of the 92 bp ITS1-5.8S-ITS2-specific qPCR product (GenBank accession number AY697929, GATACCCTTTGTGAACTTATAACCATTTTATCGTTGCCTCGGCGCTGAGCCCGGGGGGGGGTTGGCGAAGGCAGATTTTCTTCCTTCTCCCC) synthesized de novo and cloned into a plasmid pUC-GW-Kan (Azenta Life Sciences, Leipzig, Germany). Posterior regression equation analyses permitted estimating the target fragment copy number, actual number of *C. parasitica* cells or spores, in query samples depending on the mean Ct value per triplicate wells (Figure 1). For calculating the copy number in the original plasmid aliquot, the copy number was calculated using the following equation: number of copies/µL = [(6 × 10^23^) × (DNA concentration, 40 ng/µL)/molecular weight of one plasmid], where 6 × 10^23^ is the number of copies per mole, DNA concentration is given in grams per microlitre, and the molecular weight of one plasmid is in grams per mole assuming a plasmid size of 2627 bp and a 1 bp molecular weight of 660 g/mole.

During the real-time PCR from washed/crushed insect samples, the potential inhibition of the reaction via molecules present within the insect carcasses was ruled out by spiking 1 µL of pooled insects extracts with the positive controls 1:10 to 1:10,000 for the fungus.

### 2.5. Other Fungi Identification

Eppendorf tubes (2-mL) containing 1 mL of 2% Malt Extract Broth were inoculated by transferring hyphal tips (under microscope) from the edges of individual colonies. After 7 days of incubation at 25 °C, DNA was extracted using Prepman Ultra Sample Preparation Reagent (Applied Biosystems, Fisher Scientific, Horsham, UK).

DNA was amplified in 39 µL PCR reaction volume with 20 µL of GreenTaq (New England Biolabs, Hitchin, UK), 2 µL of each primer (10 µM), 14 µL molecular grade water, and 1 µL of DNA solution.

The region of the nuclear rDNA cluster, including ITS1, 5.8S rDNA gene and ITS2, was amplified with the primer pair ITS1-F (5′-CTTGGTCATTTAGAGGAAGTAA-3′) and ITS4 (5′-TCCTCCGCTTATTGATATGC-3′) [21]. Reactions were performed on a GeneAmp PCR System 9700 (Applied Biosystems, Fisher Scientific, Horsham, UK) with an initial denaturation step of 2 min at 95 °C, followed by 30 cycles of denaturation at 94 °C (35 s), annealing at 53.5 °C (55 s) and elongation at 72 °C (45 s). Final extension was for 10 min at 72 °C.

Following amplifications, PCR products were electrophoresed on 1% agarose gels at 90 V 45 min in a Wide Mini-Sub Electrophoresis System (BioRad, Watford, UK) and then digitalized and quantified with Gel Doc XR+ band quantification software in comparison with a CSL-MDNA-1 kb ladder (Cleaver Scientific, Rugby, UK). Then, amplicons were purified using the DNA clean and concentrator kit (Zymo Research, Freiburg, Germany) and sequenced by Source Bioscience Ltd. (Cambridge, UK). Forward and reverse sequences were aligned, and consensus was determined with SEQUENCHER 5.4.6. BLAST searches were conducted. All sequences obtained have been deposited in GenBank (Supplementary Table S2). Each representative isolate was deposited at the THDAS culture collection at −80 °C in glycerol 15%.

### 2.6. Other Insects’ Identification

Considering the difficulties amplifying Cytochrome Oxidase I (COI) because of the presence of the endosymbiont bacteria *Wolbachia* in all the *Torymus* sample tubes, the ITS2 region was amplified with the *Torymus* genus-specific primers ToITS2-F (5′-TGTGAACTGCAGGACACATG-3′) and ToITS2-R (5′-ATGCTTAAATTYAGCGGGTA-3′) described by Viviani et al. [22]. DNA extracts, from crushed pooled specimens using DNeasy Plant Pro kit, were used. DNA was amplified in 39 µL PCR reaction volume with each master mix unit containing 20 µL of GreenTaq (New England Biolabs), 2 µL of each primer (10 µM), 14 µL molecular grade water, and 1 µL of DNA solution. Reactions were performed with an initial denaturation step of 5 min at 95 °C, followed by 40 cycles of denaturation at 95 °C (30 s), annealing at 50 °C (40 s), and elongation at 72 °C (40 s). Final extension was done for 10 min at 72 °C. PCR products were electrophoresed, purified, sequenced, and the consensus contigs blasted as explained.

## 3. Results

### 3.1. Quantity of C. parasitica Spores

The positive spores copy number counts ranged between around 60 and around 8.5 × 10^3^. The DNA of *C. parasitica* could be quantified down to a lower detection limit magnitude of 0.004 femtograms (which corresponds to one conidium).

**Figure 2:**
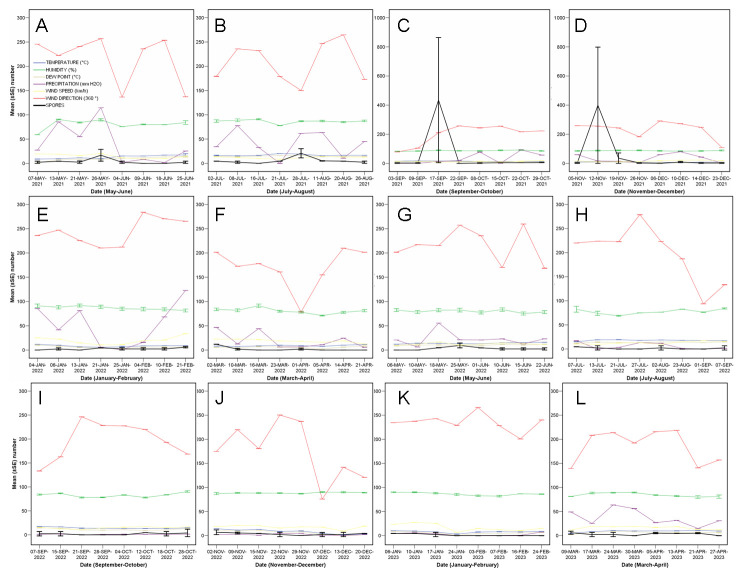
Phenology of conidia mean (±SE) number (black lines) plotted, per bimonthly period, together with all the analysed meteorological variables between May 2021 and April 2023 (**A**–**L**).

**Figure 3:**
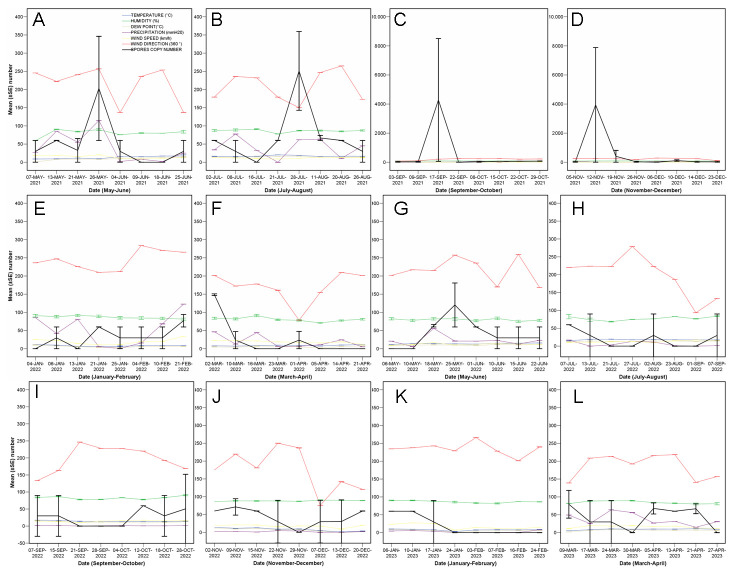
Phenology of conidia mean (±SE) ITS fragment copy number (black lines) plotted, per bimonthly period, together with all the analysed meteorological variables between May 2021 and April 2023 (**A**–**L**).

The temporal pattern showed fluctuations of peaks and troughs (Figure 2 and Figure 3), with two main peaks in the late summer and fall of 2021, and moderate peaks in spring and summer 2021. Other minor peaks were detected in winter and spring 2022, for example. This temporal effect was not statistically significant on-the-whole (conidia copy number, F = 0.976, *p* = 0.547), but, once the data were filtered retaining only those values over a threshold of 60 conidia copy number, a significant effect was detected (F = 505.53, *p* = 0.002).

The general logical correlations (Supplementary Table S3), when analysing the whole data, gave a high level of certainty to the rest of the results. Highly significant relations were detected between temperature and humidity (negative) or dew point (positive).

When doing split-plot analyses per bimonthly period, temperature, humidity, dewpoint, rainfall, wind speed, and wind duration were significantly correlated with the quantity of trapped spores at certain periods at *p* < 0.05; a positive correlation of rainfall with a significant rise in conidial load was observed in three periods (Table 1).

**Figure 4:**
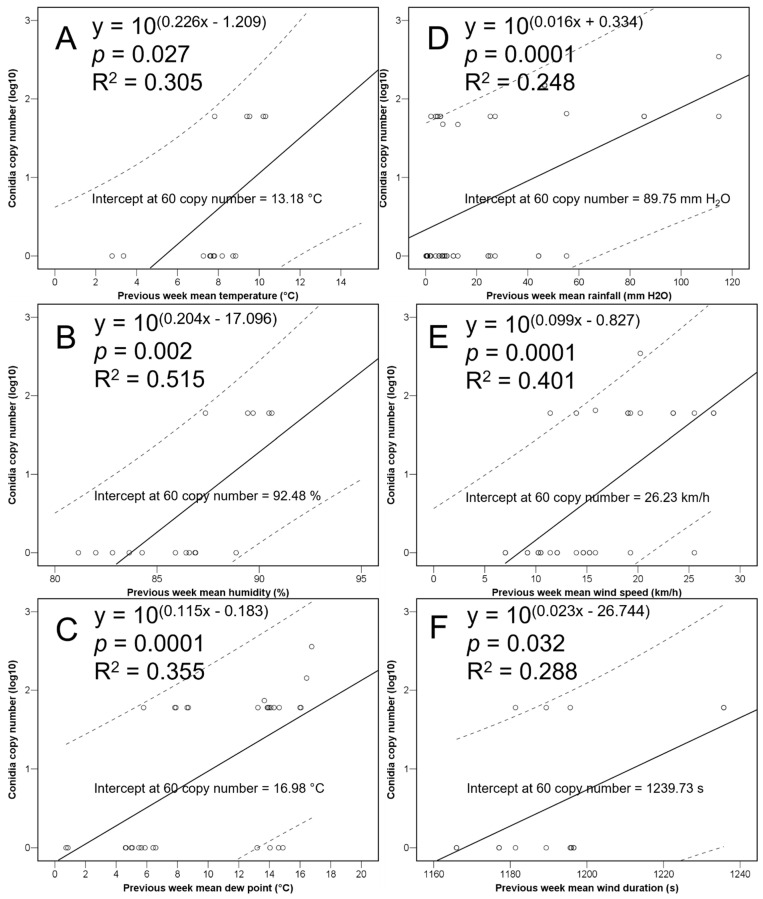
Regression equations between the conidia copy number and several previous weekly mean meteorological variables [temperature (**A**), humidity (**B**), dew point (**C**), rainfall (**D**), wind speed (**E**), and wind duration (**F**)], in the bimonthly relevant positively correlated periods, showing their significance, relation values, and respective intercepts at sixty conidia copy number threshold value. Dotted lines represent 95% confidence intervals.

The intercepts showed a threshold risk for conidia release when the weekly temperature means are over 13 °C, with rainfalls of 89 mm and winds of over 26 km/h blowing for more than 20 min. Despite the regressions being significant (Figure 4), they should be used with caution, since the R^2^ values indicate variability of the outcome variable.

### 3.2. Detection of C. parasitica from Insects

Regarding the collected insects (in total, 1243 specimens collected in propylene glycol), the dominant species in West Sussex, Devon (which presented the highest diversity), and London were, respectively, *Xylosandrus germanus*, *Anisandrus dispar*, and *Xyleborinus saxesenii*. All insect samples were tested for the presence of *C. parasitica* using real-time PCR and isolation. No samples tested positive and none yielded colonies of *C. parasitica*.

The positive controls used during the real-time PCR always amplified in the triplicate wells. The obtained three amplification curves per serial dilution point were always very tight and a difference of a couple of cycles was observed between the positive controls. The query samples as well as the negative controls never amplified except for the 18S DNA quality control. There was not inhibition by molecules present within the insect carcasses.

### 3.3. Other Fungi

Other fungi were isolated from insects (Supplementary Table S2), highlighting *Gnomoniopsis smithogilvyi* (from the OCGW), an important chestnut pathogen previously reported causing lesions on *Castanea sativa* in the UK [23], and *Daldinia concentrica* (also from the OCGW), a fungal genus with different species acting as ectosymbionts of different wasps [24]. On the other hand, examples of fungi typically associated with ambrosia beetles were *Ambrosiella grosmanniae* or *Ambrosiella hartigii*, respectively, isolated in the present study from *Anisandrus dispar* and the same plus *Xylosandrus germanus*. *Geosmithia flava*, *Haplographium penicillioides*, *Ophiostoma solheimii*, or *Sporothrix eucastanea* are also some examples of fungal species that make up the surface microbiota of bark and ambrosia beetles.

### 3.4. Other Insects

All the pooled *Torymus flavipes* samples resulted in COI-positive amplicons and sequences for the endosymbiotic bacteria *Wolbachia* [25]. The consensus ITS2 sequence for *Torymus flavipes*, common in England, was deposited in GenBank (Acc. No. OR625177).

## 4. Discussion

Quantifying the conidia (ascospores were not detected in the area) of the chestnut blight fungus and relating those quantities with temporal and climatic variables may provide information for the management of sweet chestnut tree stands, i.e., forecasting moments of high risk of new infections, then avoiding silvicultural works causing open lesions. A solid experimental design was used that had already proven its reliability in previous studies targeting *Hymenoscyphus fraxineus* [19] or *Dothistroma septosporum* [26] spores.

Based on previous articles [15,27], a high variability of conidia loads was expected, although conidia were expected to be discharged throughout the year, which was not the case. The seasonal pattern observed in England differed from that reported in France, where *C. parasitica* released spores between March and October, with a single peak during the spring [27], or that reported in Italy with two peaks, respectively, in the spring and fall [15]. It was more similar to that indicated in the USA with the main peaks occurring in late summer or fall [28], although considering the whole results in the present study, the peaks depend on the local weather conditions (if they are suitable for conidia release). In that regard, the association between temperatures and chestnut blight was investigated previously [29], reporting that temperature can modulate a certain chestnut blight outbreak. The main driver boosting the spread of *C. parasitica* in North America was air temperature [28]. Correlations between the abundance of propagules released and temperatures were also detected in France [27], as in the present study.

Regarding relative humidity, it obviously is higher when the intensity of the rainfall is also high (indeed between them correlation coefficient = 0.245, *p* = 0.001), thus explaining the positive relation between this variable and the quantity of *C. parasitica* conidia in the present study. The positive correlation with rainfall has been confirmed by other previous articles [15,27,30,31,32]. For example, Prospero and Rigling [32] reported that a rainfall event of 4.5 mm could induce an abundant sporulation of *C. parasitica*, while Lione et al. [15] recorded that the daily water input able to boost the sporulation ranges from 1 to 10 mm, which is consistent with the previous study and also in agreement with our weekly means.

Wind may be another key factor for the successful release and dissemination of airborne propagules of fungi [33,34], and indeed the current analyses showed that it did have (both its speed and duration time) a significant positive effect on the spore release of *C. parasitica*. Therefore, although the asexual conidia of this fungal species are mainly rain-splash-dispersed by raindrops within a short distance of a few metres [32], the effect of the wind speed and its duration cannot be totally ruled out. To the best of our knowledge, this is the first time relating *C. parasitica* conidia air concentrations with wind. Further studies should be directed to determine the dispersal distance ability of conidia and/or ascospores.

Extreme events such as hailstorms (not common in this geographical area) have been reported as drivers of the resurgence of chestnut blight in continental Europe because the size of the wounds on chestnut bark is positively associated with the risk of infection by *C. parasitica* [35]. In addition, spreading pathways of the propagules of *C. parasitica* other than hailstorms, winds (this study), rainfalls, humidity, and increasing temperatures have been documented, including insects and other animals. For example, fungi-eating nematodes in the genus Aphelenchoides were reported to be common in blight cankers and to act as carriers [36,37]. On the other hand, 69 insect species (mostly Coleoptera) were found harbouring the fungus [38]; bark miners (*Spulerina simploniella*) [39] and ants [40] have also been reported as carriers. Mites have been reported as carriers of the fungus [36,41,42,43,44]. The active role of mites, directly by mycelia/spores on their body or indirectly by their faecal pellets, in the spread of the chestnut blight was later emphasized [45,46,47]. In this study, we chose to target bark and ambrosia beetles only. Of the 1243 samples collected, none tested positive for *C. parasitica*, indicating that these beetles are not carriers of the fungus in England. Further work will be required to determine if the role of mites is important in the transmission of the chestnut blight fungus in England, and to compare the detection method described herein with NGS procedures using the same DNA extracts.

## 5. Conclusions

Since global warming and extreme events have been reported as drivers of the resurgence of chestnut blight, the risk of damage and losses is likely to be increased in the future. An overall increase of the temperature due to climate change could result in an expansion of the sporulation period. In general, thus, management operations should be conducted with caution, preventing injuries to the chestnut trees, especially after the occurrence of weekly periods with means over 13 °C of temperature and 89 mm of rain with winds of over 26 km/h blowing for more than 20 min. Operations are therefore likely to be less problematic after calm and dry cold weeks.

## Figures and Tables

**Table 1:** Bimonthly split-plot correlation analyses between the quantity of trapped spores (estimated either via spores serial dilutions or fragment copy number dilutions) and meteorological parameters. The dark shaded areas indicate significant correlations (*p* < 0.05) while the pale shaded areas indicate correlations with a *p* < 0.1.

		Based on Spores Serial Dilutions							Based on Fragment Copy Number Dilutions						
		Temperature (°C)	Humidity (%)	Dew Point (°C)	Rain (mm H_2_O)	Wind Speed (km/h)	Wind Direction (360°)	Wind Duration (s)	Temperature (°C)	Humidity (%)	Dew Point (°C)	Rain (mm H_2_O)	Wind Speed (km/h)	Wind Direction (360°)	Wind Duration (s)
May–Jun 2021	Pearson Correlation	−0.423	0.386	−0.181	0.629	0.493	0.185	0.183	−0.437	0.391	−0.191	0.640	0.507	0.180	0.183
	Sig. (2-tailed)	0.103	0.140	0.502	0.009	0.053	0.492	0.497	0.091	0.134	0.478	0.008	0.045	0.505	0.498
Jul–Aug 2021	Pearson Correlation	0.434	0.008	0.616	0.213	−0.036	−0.462	0.296	0.438	−0.003	0.615	0.206	−0.025	−0.462	0.306
	Sig. (2-tailed)	0.093	0.977	0.011	0.429	0.895	0.072	0.265	0.089	0.992	0.011	0.445	0.926	0.072	0.249
Sep–Oct 2021	Pearson Correlation	0.278	0.084	0.321	−0.142	−0.292	0.048	0.123	0.276	0.084	0.319	−0.141	−0.290	0.049	0.121
	Sig. (2-tailed)	0.297	0.758	0.226	0.599	0.273	0.859	0.650	0.300	0.757	0.228	0.602	0.275	0.857	0.656
Nov–Dic 2021	Pearson Correlation	0.383	0.283	0.425	−0.192	−0.313	0.115	−0.011	0.386	0.288	0.430	−0.192	−0.314	0.116	−0.011
	Sig. (2-tailed)	0.143	0.288	0.101	0.477	0.239	0.671	0.968	0.139	0.279	0.097	0.475	0.236	0.669	0.969
Jan–Feb 2022	Pearson Correlation	−0.247	−0.312	−0.268	0.014	0.212	0.118	0.474	−0.252	−0.312	−0.271	0.006	0.205	0.115	0.468
	Sig. (2-tailed)	0.356	0.239	0.316	0.960	0.430	0.664	0.064	0.347	0.239	0.309	0.983	0.447	0.672	0.067
Mar–Apr 2022	Pearson Correlation	−0.343	0.183	−0.132	0.564	0.470	0.168	−0.137	−0.348	0.182	−0.136	0.561	0.474	0.168	−0.137
	Sig. (2-tailed)	0.193	0.497	0.627	0.023	0.066	0.534	0.614	0.187	0.500	0.616	0.024	0.064	0.535	0.612
May–Jun 2022	Pearson Correlation	−0.121	0.153	0.012	0.269	−0.012	0.391	−0.423	−0.118	0.166	0.023	0.284	0.004	0.389	−0.420
	Sig. (2-tailed)	0.656	0.571	0.964	0.313	0.964	0.134	0.103	0.662	0.539	0.932	0.286	0.987	0.136	0.106
Jul–Aug 2022	Pearson Correlation	−0.400	0.390	−0.112	0.362	−0.430	0.077	0.537	−0.400	0.390	−0.112	0.362	−0.430	0.077	0.537
	Sig. (2-tailed)	0.125	0.136	0.680	0.168	0.096	0.778	0.032	0.125	0.136	0.680	0.168	0.096	0.778	0.032
Sep–Oct 2022	Pearson Correlation	0.103	0.300	0.196	0.162	0.384	−0.323	0.0001	0.104	0.292	0.193	0.159	0.383	−0.323	−0.002
	Sig. (2-tailed)	0.704	0.259	0.467	0.548	0.142	0.222	0.999	0.702	0.273	0.473	0.556	0.143	0.222	0.995
Nov–Dec 2022	Pearson Correlation	0.363	0.096	0.376	−0.128	0.345	−0.091	0.224	0.221	0.137	0.227	−0.164	0.377	−0.114	0.467
	Sig. (2-tailed)	0.167	0.723	0.151	0.637	0.191	0.737	0.403	0.449	0.625	0.416	0.559	0.166	0.687	0.079
Jan–Feb 2023	Pearson Correlation	0.552	0.718	0.654	0.508	0.810	0.099	0.321	0.552	0.718	0.654	0.508	0.810	0.099	0.321
	Sig. (2-tailed)	0.027	0.002	0.006	0.045	0.0001	0.716	0.225	0.027	0.002	0.006	0.045	0.0001	0.716	0.225
Mar–Apr 2023	Pearson Correlation	−0.247	−0.403	−0.372	−0.268	−0.244	−0.142	−0.228	−0.240	−0.401	−0.365	−0.272	−0.239	−0.135	−0.234
	Sig. (2-tailed)	0.357	0.121	0.156	0.316	0.362	0.601	0.395	0.372	0.123	0.164	0.308	0.373	0.618	0.383

**Table 2:** Number of pooled beetles of eight different species, caught in propylene glycol by ethanol-baited Lindgren funnel (West Sussex or Devon) or cross-vane (London) traps and processed for the detection of *Cryphonectria parasitica* by real-time PCR.

Date	Ambrosia Beetles																		Bark Beetles					
	WEST SUSSEX																							
	*Xylosandrus germanus*			*Anisandrus dispar*			*Xyleborinus saxesenii*			*Xyleborus dryographus*			*Trypodendron domesticum*			*Trypodendron signatum*			*Dryocoetes villosus*			*Hylesinus wachtli*		
	Gosden5	Gosden6	Hollist	Gosden5	Gosden6	Hollist	Gosden5	Gosden6	Hollist	Gosden5	Gosden6	Hollist	Gosden5	Gosden6	Hollist	Gosden5	Gosden6	Hollist	Gosden5	Gosden6	Hollist	Gosden5	Gosden6	Hollist
22/06/20	100	60	118	1		11			1				2	1	4						2			
17/07/20	52	57	96	3		10			3															
	DEVON																							
	*Xylosandrus germanus*			*Anisandrus dispar*			*Xyleborinus saxesenii*			*Xyleborus dryographus*			*Trypodendron domesticum*			*Trypodendron signatum*			*Dryocoetes villosus*			*Hylesinus wachtli*		
	upper	lower	coppice	upper	lower	coppice	upper	lower	coppice	upper	lower	coppice	upper	lower	coppice	upper	lower	coppice	upper	lower	coppice	upper	lower	coppice
21/05/21														1	2		1	3						2
07/06/21				39	80	8	2	10					17	3	12	5	3	2	1	1		7	1	2
14/06/21	1			94	60	18	7	28	5	2	3		15	3	5			1	12	2	1			
02/07/21		1		15	2	1	1						10	1	1				6	2	6			
16/07/21				7	4	3	2						2						3					
31/07/21			1	11		3	2		1				1			1								
	LONDON																							
	*Xylosandrus germanus*			*Anisandrus dispar*			*Xyleborinus saxesenii*			*Xyleborus dryographus*			*Trypodendron domesticum*			*Trypodendron signatum*			*Dryocoetes villosus*			*Hylesinus wachtli*		
	avenue	copse	woodland	avenue	copse	woodland	avenue	copse	woodland	avenue	copse	woodland	avenue	copse	woodland	avenue	copse	woodland	avenue	copse	woodland	avenue	copse	woodland
07/06/21				5		8	34	26	34															
14/06/21				2		7	23	1	18															
29/06/21						2	6	1	8															
16/07/21								1																
	Processed for *Cryphonectria* by real-time and negative.																							
	Processed for *Cryphonectria* by real-time and positive.																							

## Data Availability

The datasets generated and analysed during the current study are deposited in the FR THDAS repository and are available upon reasonable request.

## Supplementary Materials

The following supporting information can be downloaded at: https://www.mdpi.com/article/10.3390/jof10030181/s1, Table S1: Total number of pooled beetles identified and processed for *Cryphonectria parasitica* real-time PCR; Table S2: Isolated and identified fungal species from three alive dry-collected ambrosia beetle species, one bark beetle, and two hyper parasitic wasps’ species, with the originals’ or respective dilutions’ positive culture isolations distribution indicated by black shaded areas. Note: crushed insects only rendered bacteria that were not identified at the species level; Table S3: General correlation analyses between the quantity of trapped spores (estimated either via spore serial dilutions or fragment copy number dilutions) and meteorological parameters. The dark shaded areas indicate significant correlations (*p* < 0.05).
