# Supplementary material for: Forecasting of Airborne Conidia Quantities and Potential Insect Associations of Cryphonectria parasitica, the Causal Agent of Chestnut Blight, in England
Source: J Fungi (Basel). 2024 Feb 28;10(3):181. doi: 10.3390/jof10030181 (PMC10971521; doi:10.3390/jof10030181)
Supplement: Supplementary file 1 [file jof-10-00181-s001.zip › jof-2890234-SI.pdf]

Journal of *Fungi* MDPI

Supplementary Table S1. Total number of pooled beetles identified and processed for *Cryphonectria parasitica* real-time.

**Insect species**	**West Sussex**	**Devon**	**London**	**Total**
**Ambrosia beetles**				
Xylosandrus germanus	483	3	0	486
Anisandrus dispar	25	345	24	394
Xyleborinus saxesenii	4	58	152	214
Xyleborus dryographus	0	5	0	5
Trypodendron domesticum	7	73	0	80
Trypodendron signatum	0	16	0	16
**Bark beetles**				
Dryocoetes villosus	2	34	0	36
Hylesinus wachtli	0	12	0	12
**Total**	521	546	176	1,243

Journal of *Fungi*

MDPI

Supplementary Table S2. Isolated and identified fungal species from alive dry-collected three ambrosia beetle species, one bark beetle, and two hyper parasitic wasps' species, with indication of the originals or respective dilutions positive culture isolations distribution indicated by black shaded areas. Note: Crushed insects only rendered bacteria that were not identified to species level.

			AMBROSIA BEETLES																		BARK BEETLES						HYMENOPTERA											
		Acc. No.	*Xylosandrus germanus*						*Anisandrus dispar*						*Xyleborinus saxeseni*						*Dryocoetes villosus*						*Dryocosmus kuriphilus*						*Torymus flavipes*					
			1	1	1:10	1:10	1:100	1:100	1	1	1:10	1:10	1:100	1:100	1	1	1:10	1:10	1:100	1:100	1	1	1:10	1:10	1:100	1:100	1	1	1:10	1:10	1:100	1:100	1	1	1:10	1:10	1:100	1:100
	Fungal taxon		R1	R2	R1	R2	R1	R2	R1	R2	R1	R2	R1	R2	R1	R2	R1	R2	R1	R2	R1	R2	R1	R2	R1	R2	R1	R2	R1	R2	R1	R2	R1	R2	R1	R2	R1	R2
1	*Acremonium sclerotigenum*	OK661035														■																						
2	*Alternaria infectoria*	OK661036	■	■	■	■	■	■															■	■	■	■												
3	*Ambrosiella grossmanniae*	OK661037							■	■			■																									
4	*Ambrosiella hartigii*	OK661038	■	■	■				■	■	■		■																									
5	*Aureobasidium pullulans*	OK661039	■	■		■																					■	■		■		■						
6	*Botrytis cinerea*	OK661040				■																																
7	*Cladosporium* cf. *allicinum*	OK661041																									■	■					■					
8	*Cladosporium cladosporioides*	OK661042	■	■	■	■	■	■							■																							
9	*Cladosporium* cf. *sloanii*	OK661043																											■	■	■	■						
10	*Cryptocline actostaphyli*	OK661044																									■											
11	*Daldinia concentrica*	OK661045																									■											
12	*Fusarium avenaceum*	OK661046	■	■	■		■	■																	■	■												
13	*Geosmithia flava*	OK661047				■																																
14	*Gnomoniopsis smithogilvyi*	OK661048																										■										
15	*Haplographium penicillioides*	OK661049	■	■	■	■	■	■																														
16	*Metapochonia bubillosa*	OK661050													■	■																						
17	*Mortierella gamsii*	OK661051	■	■								■																										
18	*Ophiostoma solheimii*	OK661052								■																												
19	*Paraphaeosphaeria neglecta*	OK661053					■																															
20	*Penicillium bialowizense*	OK661054						■																														
21	*Penicillium brevicompactum*	OK661055		■																																		

Supplementary Table S2. Continued.

			AMBROSIA BEETLES																		BARK BEETLES						HYMENOPTERA											
		Acc. No.	*Xylosandrus germanus*						*Anisandrus dispar*						*Xyleborinus saxeseni*						*Dryocoetes villosus*						*Dryocosmus kuriphilus*						*Torymus flavipes*					
			1	1	1:10	1:10	1:100	1:100	1	1	1:10	1:10	1:100	1:100	1	1	1:10	1:10	1:100	1:100	1	1	1:10	1:10	1:100	1:100	1	1	1:10	1:10	1:100	1:100	1	1	1:10	1:10	1:100	1:100
	Fungal taxon		R1	R2	R1	R2	R1	R2	R1	R2	R1	R2	R1	R2	R1	R2	R1	R2	R1	R2	R1	R2	R1	R2	R1	R2	R1	R2	R1	R2	R1	R2	R1	R2	R1	R2	R1	R2
22	*Penicillium charlesii*	OK661056	■	■	■																																	
23	*Penicillium cosmopolitanum*	OK661057	■	■	■	■	■	■													■		■	■	■	■	■	■	■	■	■	■						
24	*Penicillium manginii*	OK661058																■																				
25	*Penicillium sajarovii*	OK661059					■	■													■		■	■	■	■												
26	*Penicillium soppi*	OK661060	■		■		■																															
27	*Penicillium spathulatum*	OK661061	■		■		■																				■	■	■	■	■	■						
28	*Penicillium spinulosum*	OK661062	■		■		■																															
29	*Penicillium ubiquetum*	OK661063	■		■		■																															
30	*Periconia sp.*	OK661064	■	■	■	■																																
31	*Pichia gilliermondii*	OK661065																										■										
32	*Sporothrix eucastanea*	OK661066												■																								
33	*Trichoderma atroviride*	OK661067	■		■																				■	■												
34	*Trichoderma lixii*	OK661068							■	■	■	■																					■	■				
35	*Trichoderma koningiopsis*	OK661069					■																															
36	*Trichoderma sp.*	OK661070														■		■	■																			
37	*Umbelopsis isabellina*	OK661071									■			■																								
38	*Umbelopsis ramanniana*	OK661072			■	■	■																															
39	*Xenoacremonium falcatum*	OK661073															■	■	■																			

Supplementary Table S3. General correlation analyses between the quantity of trapped spores (estimated either via spore serial dilutions or fragment copy number dilutions) and meteorological parameters. The dark shaded areas indicate significant correlations ($p < 0.05$).

		**Spores number**	**Copy number**	**Temperature (°C)**	**Humidity (%)**	**Dew point (°C)**	**Rain (mm $H_2$0)**	**Wind speed (km/h)**	**Wind direc. (360 °)**	**Wind duration (s)**	**Moon phase** [*]
Spores number	Pearson Correlation	1	1	0.046	0.108	0.079	-0.024	-0.108	0.056	0.030	0.095
	Sig. (2-tailed)		0.0001	0.530	0.139	0.276	0.747	0.139	0.445	0.678	0.192
Copy number	Pearson Correlation		1	0.046	0.111	0.081	-0.020	-0.106	0.056	0.031	0.094
	Sig. (2-tailed)			0.531	0.127	0.271	0.784	0.147	0.443	0.676	0.196
Temperature (°C)	Pearson Correlation			1	-0.272	0.955	-0.112	-0.370	-0.033	0.039	0.009
	Sig. (2-tailed)				0.0001	0.0001	0.122	0.0001	0.652	0.592	0.904
Humidity (%)	Pearson Correlation				1	0.020	0.245	0.166	0.020	0.050	0.023
	Sig. (2-tailed)					0.783	0.001	0.022	0.786	0.492	0.748
Dew point (°C)	Pearson Correlation					1	-0.042	-0.328	-0.026	0.060	0.017
	Sig. (2-tailed)						0.567	0.0001	0.723	0.408	0.812
Rain (mm $H_2$0)	Pearson Correlation						1	0.382	0.314	-0.004	0.007
	Sig. (2-tailed)							0.0001	0.0001	0.958	0.921
Wind speed (km/h)	Pearson Correlation							1	0.089	0.039	-0.049
	Sig. (2-tailed)								0.221	0.593	0.499
Wind direc. (360 °)	Pearson Correlation								1	-0.100	0.170
	Sig. (2-tailed)									0.171	0.019
Wind duration (s)	Pearson Correlation									1	-0.176
	Sig. (2-tailed)										0.015
